# PubChem3D: Biologically relevant 3-D similarity

**DOI:** 10.1186/1758-2946-3-26

**Published:** 2011-07-22

**Authors:** Sunghwan Kim, Evan E Bolton, Stephen H Bryant

**Affiliations:** 1 National Center for Biotechnology Information, National Library of Medicine, National Institutes of Health, Department of Health and Human Services, 8600 Rockville Pike, Bethesda, MD 20894, USA

## Abstract

**Background:**

The use of 3-D similarity techniques in the analysis of biological data and virtual screening is pervasive, but what is a biologically meaningful 3-D similarity value? Can one find statistically significant separation between "active/active" and "active/inactive" spaces? These questions are explored using 734,486 biologically tested chemical structures, 1,389 biological assay data sets, and six different 3-D similarity types utilized by PubChem analysis tools.

**Results:**

The similarity value distributions of 269.7 billion unique conformer pairs from 734,486 biologically tested compounds (all-against-all) from PubChem were utilized to help work towards an answer to the question: what is a biologically meaningful 3-D similarity score? The average and standard deviation for the six similarity measures *ST*^*ST-opt*^, *CT*^*ST-opt*^, *ComboT*^*ST-opt*^, *ST*^*CT-opt*^, *CT*^*CT-opt*^, and *ComboT*^*CT-opt *^were 0.54 ± 0.10, 0.07 ± 0.05, 0.62 ± 0.13, 0.41 ± 0.11, 0.18 ± 0.06, and 0.59 ± 0.14, respectively. Considering that this random distribution of biologically tested compounds was constructed using a single theoretical conformer per compound (the "default" conformer provided by PubChem), further study may be necessary using multiple diverse conformers per compound; however, given the breadth of the compound set, the single conformer per compound results may still apply to the case of multi-conformer per compound 3-D similarity value distributions. As such, this work is a critical step, covering a very wide corpus of chemical structures and biological assays, creating a statistical framework to build upon.

The second part of this study explored the question of whether it was possible to realize a statistically meaningful 3-D similarity value separation between reputed biological assay "inactives" and "actives". Using the terminology of noninactive-noninactive (NN) pairs and the noninactive-inactive (NI) pairs to represent comparison of the "active/active" and "active/inactive" spaces, respectively, each of the 1,389 biological assays was examined by their 3-D similarity score differences between the NN and NI pairs and analyzed across all assays and by assay category types. While a consistent trend of separation was observed, this result was not statistically unambiguous after considering the respective standard deviations. While not all "actives" in a biological assay are amenable to this type of analysis, *e.g.*, due to different mechanisms of action or binding configurations, the ambiguous separation may also be due to employing a single conformer per compound in this study. With that said, there were a subset of biological assays where a clear separation between the NN and NI pairs found. In addition, use of combo Tanimoto (ComboT) alone, independent of superposition optimization type, appears to be the most efficient 3-D score type in identifying these cases.

**Conclusion:**

This study provides a statistical guideline for analyzing biological assay data in terms of 3-D similarity and PubChem structure-activity analysis tools. When using a single conformer per compound, a relatively small number of assays appear to be able to separate "active/active" space from "active/inactive" space.

## Background

Recent advances in combinatorial chemistry [1-6] and high-throughput screening technology [7-17] have made the synthesis and screening of diverse chemical compounds easier, helping to create a demand in the biomedical research community for archives of publicly available screening data. To help satisfy this demand, the U.S. National Institutes of Health launched the PubChem project (http://pubchem.ncbi.nlm.nih.gov) [18-21] as a part of its Molecular Libraries Roadmap Initiative. PubChem archives contributed biological screening data and chemical information from various data sources in academia and industry, and offers its contents free of charge to biomedical researchers, helping to facilitate scientific discovery.

PubChem consists of three primary databases: Substance, Compound, and BioAssay. While the PubChem Substance database (unique identifier SID) contains information provided by individual depositors, the PubChem Compound database (unique identifier CID) contains the unique standardized chemical structure contents extracted from the PubChem Substance database. PubChem provides various analysis tools to relate chemical structures to the biological activity data stored in the PubChem BioAssay database (unique identifier AID).

The PubChem3D project [22-25], launched, in part, to help users identify useful structure-activity relationships, generates a theoretical 3-D conformer model [22,23] for each molecule in the PubChem Compound database, whenever it is possible. An all-against-all 3-D neighboring relationship (known as "Similar Conformers") [24] is pre-computed to help users to locate related data in the archive, augmenting the complementary "Similar Compounds" relationship, based on 2-D similarity of the PubChem subgraph binary fingerprint [26].

PubChem3D uses two 3-D similarity measures: shape-Tanimoto (ST) [24,27-30] and color-Tanimoto (CT) [24,27,28]. The ST score is a measure of shape similarity, which is defined as the following:(1)

where *V*_*AA *_and *V*_*BB *_are the self-overlap volume of conformers A and B and *V*_*AB *_is the common overlap volume between them. The CT score, given by **Equation (2)**, quantifies the similarity of 3-D orientation of functional groups used to define pharmacophores (henceforth referred to simply as "features") between conformers by checking the overlap of fictitious "color" atoms [28] used to represent the six functional group types: hydrogen-bond donors, hydrogen-bond acceptors, cation, anion, hydrophobes, and rings.(2)

where, the index "*f*" indicates any of the six independent fictitious feature atom types,  and  are the self-overlap volumes for feature atom type *f *and  is the overlap volume of conformers A and B for feature atom type *f*. The ST and CT scores range between 0 (for no similarity) and 1 (for identical molecules). These similarity metrics can be combined to create a Combo-Tanimoto (ComboT), as specified by **Equation (3)**:(3)

The ST and CT similarity metrics attempt to cover key aspects important for locating chemical structures that may have similar biological activity. ST helps to identify molecules that can adopt a particular 3-D shape, *e.g.*, of an inhibitor bound in a particular conformational orientation in a protein binding pocket. Considering that a hydrocarbon and a drug molecule could adopt the same shape, CT helps to identify molecules with similar 3-D orientation of features, *e.g.*, necessary for making binding interactions between a small molecule and protein binding pocket. This suggests that two molecules with highly similar 3-D shape and 3-D feature orientations may also have similar biological activity. It should be no small wonder that such similarity metrics have garnered widespread use in virtual screening [31,32]. It leads one to wonder: what is a statistically meaningful 3-D similarity score? Or, in other words, if one was to examine the 3-D similarities between biologically tested compounds, what does the distribution look like? In the case of 2-D similarity, one only needs the molecule graph to make a comparison but, in the case of 3-D similarity, molecules can potentially adopt a number of different conformations. Is it sufficient to use only a single conformer per compound and still realize a statistically meaningful difference or separation between the 3-D similarities of reputed actives and inactives from a biological test?

In the present paper, two important questions concerning ST, CT, and ComboT as 3-D similarity measures are investigated. The first question is "if we randomly select any two conformers from the PubChem Compound database, what values of ST, CT, and ComboT scores will be expected on the average?" With knowledge of these values, one can evaluate a statistical significance of the similarity score between any two conformers in PubChem (*e.g.*, if their similarity score becomes greater than what one expects for a random conformer pair, it may be statistically more meaningful).

The second question we seek to answer in this study is "for a given bioassay in PubChem, what is the average difference in similarity scores between the noninactive-noninactive (NN) pairs and the noninactive-inactive (NI) pairs, when a single conformer per compound is used for 3-D similarity computation?" The choice of terminology of NN and NI are necessary considering that the definition of an "active" is not always specified in PubChem. Therefore, for the purposes of this study, we consider "active space" to be anything not specified to be "inactive", thus the term "noninactive" is used in place of "active". This may help provide users with an idea on the separation in the 3-D shape and feature spaces between the active and inactive compounds tested in a given bioassay. An additional question we will answer is: does an optimization type affect the similarity scores? Currently, the PubChem 3-D neighboring involves a shape superposition optimization that maximizes the ST scores [24], but it may be possible to optimize a feature superposition that maximizes the CT score. Will the ST-optimization and CT-optimization make any changes in a 3-D similarity-based bioassay data analysis?

## Results and Discussion

### A. Notations

In the present study, we consider six different similarity measures: ST, CT, and ComboT for two different optimization types (either ST-optimized or CT-optimized). They are denoted with a superscript, which represents the optimization type (either "ST-opt" or "CT-opt"), and a subscript, which specifies the type of CID pairs ("NN" for the NN pairs and "NI" for the NI pairs). The subscript "NN-NI" is used for the similarity score difference between the NN and NI pairs. For example,  and  indicate the ST-optimized ComboT scores for the NN and NI pairs, respectively, while  means the difference between the two. The word "XT" is used when we refer to any of the similarity measures (*i.e., ST, CT, and ComboT*), or a similarity score in a general sense.

In the second part of this study, we analyze the average and standard deviation of the similarity scores *of CID pairs for a given AID*, and these per-AID average and standard deviation are denoted with Greek letters μ and σ, respectively, followed by the corresponding similarity measure in parentheses [*e.g.*,  and ]. The per-AID average and standard deviation of the similarity score difference between the NN and NI pairs for a given AID are computed using the following equations:(4)(5)

where XT is one of the six similarity measures (*i.e.*, *ST*^*ST-opt*^, *CT*^*ST-opt*^, *ComboT*^*ST-opt*^, *ST*^*CT-opt*^, *CT*^*CT-opt*^, and *ComboT*^*CT-opt*^), and *n*_*NN *_and *n*_*NI *_are the number of the NN pairs and NI pairs for the AID, respectively. When we refer to the average and standard deviation of the per-AID statistical parameters *over a set of AIDs*, we use additional Greek letters μ and σ, respectively, followed by the corresponding statistical parameter in brackets. For example,  and  represent the overall average and standard deviation of  over a set of AIDs, while  and  indicate the overall average and standard deviation of .

### B. 3-D similarity score distribution of random conformer pairs

#### B-1. Structural and chemical characteristics of the biologically tested molecules

**Figure 1:**
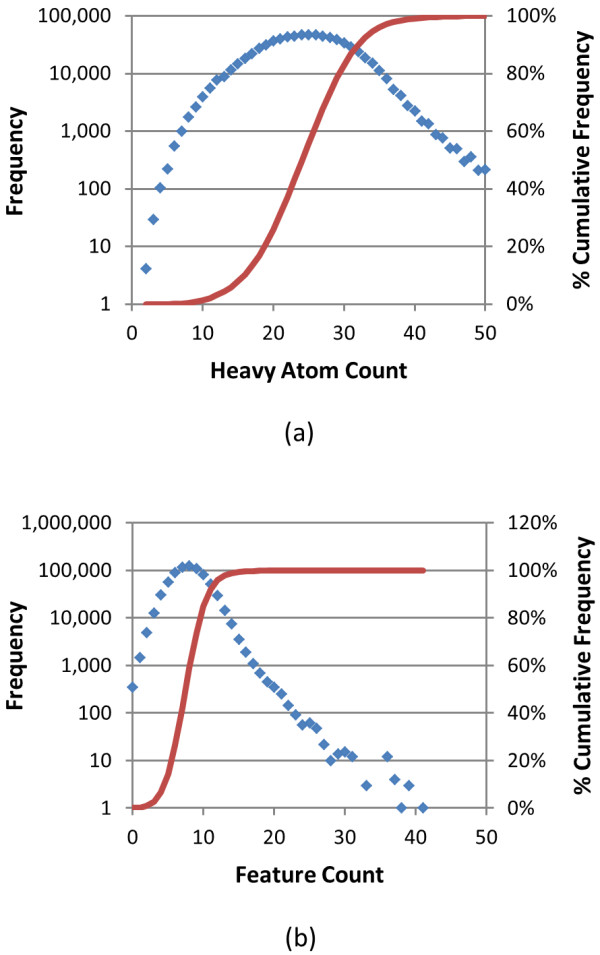
**Atom and feature count histograms of biologically tested compounds**. Frequency (blue) and percent cumulative frequency (red) of (a) heavy atom count and (b) total feature count for the 734,486 molecules tested in at least one bioassay archived in the PubChem BioAssay database.

**Figure 2:**
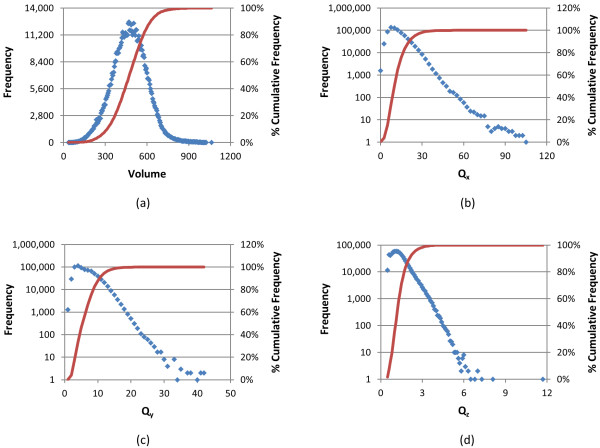
**Conformer volume and quadrupole histograms of biologically tested compounds**. Frequency (blue) and percent cumulative frequency (red) of (a) volume, (b) Q_x_, (c) Q_y_, and (d) Q_z _for the 734,486 molecules tested in at least one bioassay archived in the PubChem BioAssay database.

**Figure 3:**
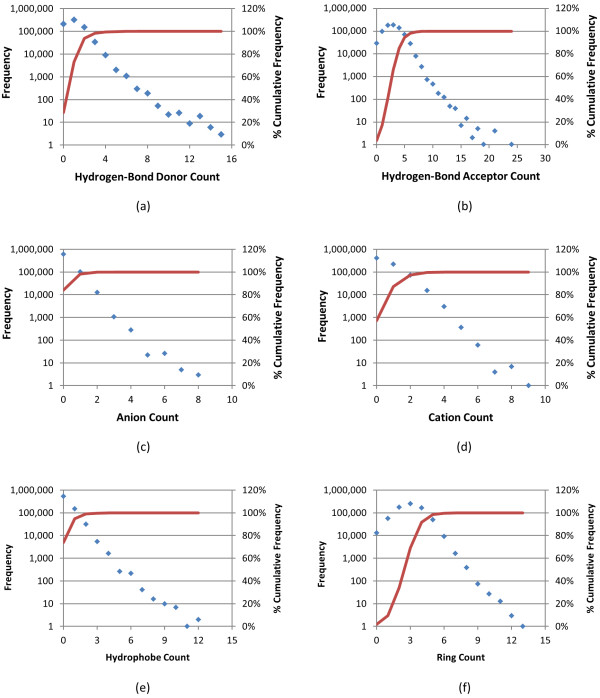
**Individual feature histograms of biologically tested compounds**. Frequency (blue) and percent cumulative frequency (red) of respective feature atom count for the 734,486 molecules tested in at least one bioassay archived in the PubChem BioAssay database: (a) hydrogen-bond donor count, (b) hydrogen-bond acceptor count, (c) anion count, (d) cation count, (e) hydrophobe count, and (f) ring count.

As of January 2010, the PubChem BioAssay database had 2,008 bioassay records, (ranging from AID 1 to AID 2310) and 734,486 molecules with a 3-D conformer model were tested in at least one of these bioassays. The structural and chemical characteristics of these biologically tested molecules are shown in Figures 1, 2 and 3, and they are compared with those of the entire PubChem3D contents (26,157,365 CIDs as of September 2010) in Table 1. The average and standard deviation of the heavy atom count per-CID are 24.6 ± 6.4, slightly less than those across the entire PubChem3D contents (26.3 ± 7.0). The conformer monopole volume (V) and three components of the shape quadrupole moments (Q_x_, Q_y_, and Q_z_, which give a sense of the conformer length, width, and height dimensions, respectively) [25] of the biologically tested molecules default conformer are also slightly less than those across the entire PubChem3D contents (474.1 ± 124.0 Å^3 ^*vs. *509.0 ± 137.1 Å^3 ^for V, 12.6 ± 7.0 Å^5 ^*vs. *13.6 ± 7.8 Å^5 ^for Q_x_, 3.3 ± 1.6 Å^5 ^*vs. *3.6 ± 1.8 Å^5 ^for Q_y_, 1.3 ± 0.6 Å *vs. *1.5 ± 0.6 Å^5 ^for Q_z_). As shown in Figure 1(b) and Table 1, the 734,486 biologically tested molecules have 8.1 ± 2.6 features on average, slightly less than the entire PubChem3D contents does (8.5 ± 2.7). The count for each of the six feature types of the biologically tested molecules is equal to or slightly less than those of the entire PubChem3D contents.

**Table 1:** Summary statistics of chemical structure descriptors.

	Biologically Tested Compounds (734,486 CIDs)	Entire PubChem3D Contents (26,157,365 CIDs)
Heavy atom count	24.6 ± 6.4	26.3 ± 7.0
Monopole volume (Å^3^)	474.1 ± 124.0	509.0 ± 137.1
Q_x _(Å^5^)	12.6 ± 7.0	13.6 ± 7.8
Q_y _(Å^5^)	3.3 ± 1.6	3.6 ± 1.8
Q_z _(Å^5^)	1.3 ± 0.6	1.5 ± 0.6
Total feature count	8.1 ± 2.6	8.5 ± 2.7
Hydrogen-bond acceptor count	2.9 ± 1.6	3.0 ± 1.6
Hydrogen-bond donor count	1.1 ± 1.0	1.2 ± 1.0
Anion count	0.2 ± 0.4	0.2 ± 0.4
Cation count	0.6 ± 0.8	0.7 ± 0.9
Hydrophobe count	0.3 ± 0.6	0.5 ± 0.8
Ring count	3.0 ± 1.2	3.0 ± 1.3

The average and standard deviation of heavy atom count, monopole volume, quadrupole components, and feature counts (by total and each of the six feature types) for 734,486 CIDs biologically tested compounds considered in this study and those for the entire PubChem3D contents (26,157,365 CIDs as of September 2010).

#### B-2. Distribution of 3-D similarity scores for biologically tested molecules

One key question this study attempts to answer is: what are statistically meaningful 3-D similarity values for biologically tested molecules? By using the entire set of 734,486 biologically tested molecules in PubChem (as of late January 2010) and their 269,734,474,855 unique CID pairs, we believe this to be a sufficient corpus to make such a determination in a general sense. What may be questionable (to some) is the intention to use only a single conformer per compound for each of the CID pairs.

The reasons for this choice are rather practical. The use of two diverse conformers per compound yields four times more unique conformer pairs and using three diverse conformers per compound makes the unique conformer pair set nine times larger and so on. In other words, the problem size scales as a square of the conformers per compound considered. We could sample the 734,486 compounds into a smaller set, to say ten percent of the original dataset and then consider three diverse conformers per compound to yield approximately the same count of conformer pairs, but are three diverse conformers per compound sufficient? If we down sampled to 1% of the biologically tested compounds and used ten diverse conformers per compound, would ten diverse conformers per compound be sufficient and would the random 1% of the compound set be sufficient to represent biologically tested compounds? For the purposes of this study, we will ignore the multiple conformer representation issue and consider a single conformer per compound to be sufficiently random to provide a useful set of statistically meaningful 3-D similarity thresholds; however, a more detailed study may be necessary to determine the full effect of using multiple conformers per compound, *e.g.*, when picking the best conformer pair per compound pair.

**Figure 4:**
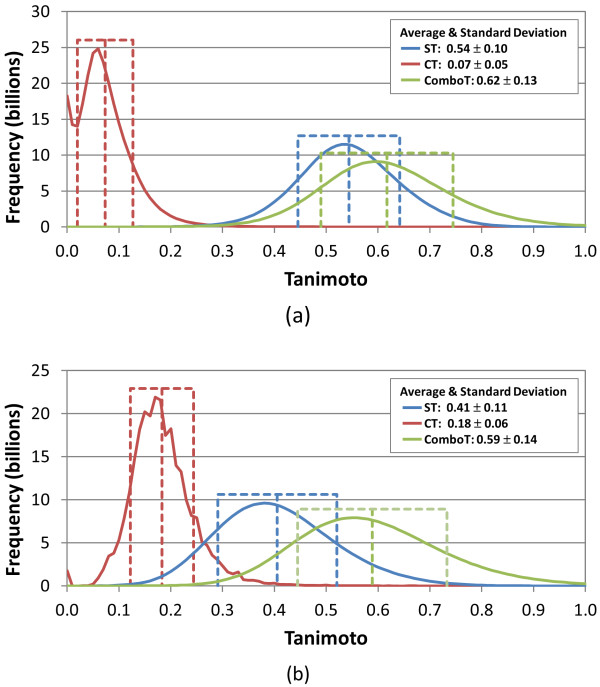
**Overall 3-D similarity statistics between biologically tested compounds**. Distribution of 3-D similarity scores of 269,734,474,855 conformer pairs, arising from the 734,486 molecules tested in at least one bioassay archived in the PubChem BioAssay database: (a) ST-optimized similarity scores and (b) CT-optimized similarity scores. A single conformer was used for each compound. All values binned in 0.01 increments.

To investigate the average values of ST, CT, and ComboT for random conformer pairs, we downloaded all 734,486 biologically tested molecules from PubChem that had a theoretical 3-D description, and the six similarity scores [*i.e.*, *ST*^*ST-opt*^, *CT*^*ST-opt*^, *ComboT*^*ST-opt*^, *ST*^*CT-opt*^, *CT*^*CT-opt*^, and *ComboT*^*CT-opt*^] were computed for all 269,734,474,855 unique CID pairs arising from all possible combination of the 734,486 CIDs, using a single conformer per-CID. The distribution of these scores represents the 3-D similarity scores one would get from any two conformers randomly selected from the PubChem database. The distributions of the similarity scores, binned in 0.01 increments, are shown in Figure 4 and their statistics are summarized in Table 2. The average and standard deviation for *ST*^*ST-opt*^, *CT*^*ST-opt*^, *ComboT*^*ST-opt*^, *ST*^*CT-opt*^, *CT*^*CT-opt*^, and *ComboT*^*CT-opt *^were 0.54 ± 0.10, 0.07 ± 0.05, 0.62 ± 0.13, 0.41 ± 0.11, 0.18 ± 0.06, and 0.59 ± 0.14, respectively. The conformer pairs whose similarity scores are equal to or smaller than μ+σ account for 85% to 87% of the 269.7 billion CID pairs, and the corresponding fractions for the μ+2σ threshold range from 96% to 98%. This information may be used to evaluate the statistical significance of the similarity score between any two conformers. For example, if the *ST*^*ST-opt *^value between two conformers is 0.74, the probability of randomly getting a *ST*^*ST-opt *^score equal to or higher than 0.74 is only 2%, and hence, one may consider that the two conformers have statistically meaningful similarity in terms of *ST*^*ST-opt*^.

**Table 2:** Summary statistics for 3-D similarity over all biologically tested compounds.

					**% Cumulative pair count**^***a***^	
	Mean (μ)	Std. Dev.(σ)	μ + σ	μ + 2σ		
					μ + σ	μ + 2σ
ST^ST-opt^	0.54	0.10	0.64	0.74	85%	98%
CT^ST-opt^	0.07	0.05	0.12	0.17	86%	96%
ComboT^ST-opt^	0.62	0.13	0.75	0.88	87%	97%
ST^CT-opt^	0.41	0.11	0.52	0.63	85%	97%
CT^CT-opt^	0.18	0.06	0.24	0.30	87%	96%
ComboT^CT-opt^	0.59	0.14	0.73	0.87	85%	96%

Statistics of the similarity scores for 269,734,474,855 conformer pairs arising from all unique combinations of 734,486 CIDs tested in at least one bioassay archived in the PubChem BioAssay database. ^*a *^% Cumulative count of the conformer pairs whose similarity scores are equal to or less than μ + σ or μ + 2σ.

Note that the PubChem "Similar Conformers" 3-D neighboring requires the *ST*^*ST-opt *^≥ 0.8 and *CT*^*ST-opt *^≥ 0.5 for two molecules to become neighbors of each other. The conformer pairs whose ST value is smaller than 0.80 correspond to 99.32% of the random ST score distribution. Similarly, the conformer pairs with *CT*^*ST-opt *^< 0.50 correspond to 99.98% of the random CT score distribution. Therefore, if the *ST*^*ST-opt *^and *CT*^*ST-opt *^scores are assumed to be independent of each other, the probability of two conformers being identified as 3-D "Similar Conformers" neighbors of each other by chance is (100 - 99.32) × (100 - 99.98) = 0.0136% (or 1 in 7,353). Note that the *CT*^*ST-opt *^score is not completely independent of the *ST*^*ST-opt *^score because it is evaluated at the ST-optimized alignment. Therefore, the probability of random conformers being identified as PubChem 3-D neighbors will be higher than the estimated value of 0.0136%, but it will still be smaller than 1%.

**Figure 5:**
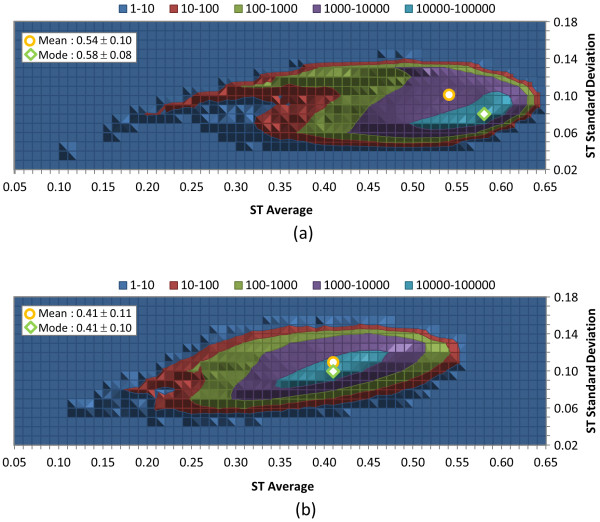
**Per-CID shape similarity statistics of biologically tested compounds**. Distribution of the average and standard deviation of the ST scores for each of the 734,486 molecules tested in at least one bioassay archived in the PubChem BioAssay database: (a) ST-optimized ST (*ST*^*ST-opt*^) and (b) CT-optimized ST (*ST*^*CT-opt*^). All values binned in 0.01 increments.

**Figure 6:**
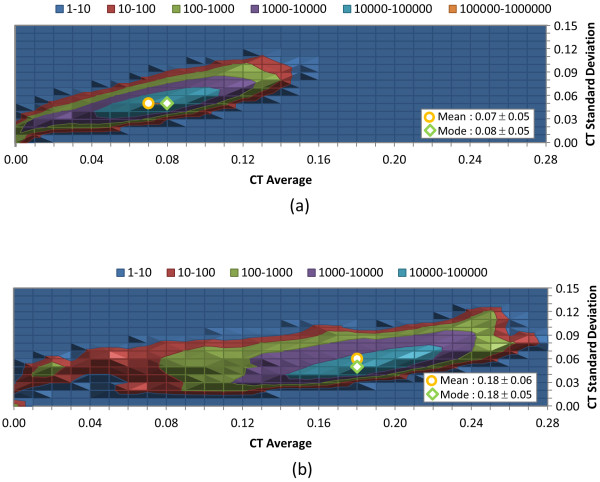
**Per-CID feature similarity statistics of biologically tested compounds**. Distribution of the average and standard deviation of the CT scores for each of 734,486 molecules tested in at least one bioassay archived in the PubChem BioAssay database: (a) ST-optimized CT (*CT*^*ST-opt*^) and (b) CT-optimized CT (*CT*^*CT-opt*^). All values binned in 0.01 increments.

**Figure 7:**
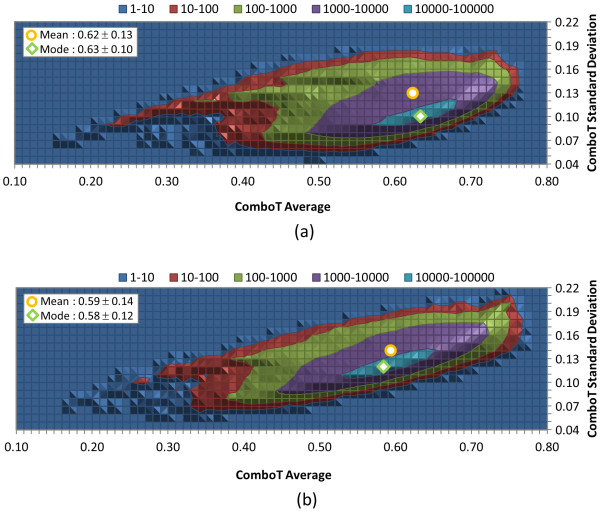
**Per-CID shape plus feature similarity statistics of biologically tested compounds**. Distribution of the average and standard deviation of the ComboT scores for each of the 734,486 molecules tested in at least one bioassay archived in the PubChem BioAssay database: (a) ST-optimized ComboT (*ComboT*^*ST-opt*^) and (b) CT-optimized ComboT (*ComboT*^*CT-opt*^). All values binned in 0.01 increments.

Figures 5, 6, and 7 show the distribution of the average and standard deviation of the 3-D similarity scores per-CID (computed from the similarity scores between one CID of the 734-K conformer set and all the other conformers in the set) for ST, CT, and ComboT for both ST-optimized and CT-optimized superpositions, representing the similarity scores that one may expect when a conformer in PubChem is compared with a randomly selected conformer. Most conformers have the average and standard deviation similar to those for the random conformers listed in Table 2. However, in the case of *ST*^*ST-opt *^[Figure 5 (a)] there is a bit of skew in the distribution of average ST value per CID towards the maximum value, peaking at 0.58, as opposed to the overall average of 0.54. Also of interest in Figure 5 (a), the ST average per-CID rapidly drops off as the ST average approaches 0.65. Note that a small fraction of biologically tested CIDs in PubChem have low average similarity scores per-CID, which indicates their relative uniqueness in the 3-D shape space (*i.e.*, their 3-D shape and/or feature orientations may be very different from most biologically tested molecules in PubChem, resulting in low similarity scores on average).

Potentially surprising when looking at feature similarity statistics in Table 2 is that standard deviation values for CT are about half that found for ST. When looking at the per-CID statistics in Figure 6, one sees that the range of standard deviation of CT is comparable to that of ST, although with a significant population of CIDs on the lower end of the standard deviation. Why is this so? Presumably, the 3-D orientation of features is substantially more diverse than the 3-D molecular shape, keeping both the average and standard deviation values low when compared to all other biologically tested compounds.

An important observation is that the overall *ComboT*^*ST-opt *^and *ComboT*^*CT-opt *^scores have very similar average values, as shown in Table 2. Whereas the *ST*^*ST-opt *^average was greater by 0.13 than the *ST*^*CT-opt *^average, the CT-optimization results in an average *CT*^*CT-opt *^score greater by 0.11 than that of *CT*^*ST-opt*^. As a result, the difference in averages between *ComboT*^*ST-opt *^and *ComboT*^*CT-opt *^were only 0.03, implying that the ComboT score is not very sensitive to the type of optimization. A similar optimization-type dependency of the ST, CT, and ComboT scores was observed in Figures 5, 6 and 7. That is, whereas the ST-optimization results in an increased ST and decreased CT scores, the CT-optimization gives a decreased ST and increased CT scores, resulting in the average ComboT score that is relatively constant regardless of the optimization type employed. However, as shown in Figure 7, the *ComboT*^*CT-opt *^data had a narrower range of standard deviation variation per-CID than *ComboT*^*ST-opt *^and the standard deviation for *ComboT*^*CT-opt *^per-CID appeared to linearly increase as a function of the per-CID average value.

### C. 3-D similarity score differences for the NN and NI pairs

The second part of this study examines the question: is it sufficient to only use a single conformer per compound and still realize a statistically meaningful difference or separation between the 3-D similarities of reputed actives and inactives? Or, to say this in another way, are noninactive and inactive compounds in a given bioassay well separated in 3-D shape/feature space? If so, one would expect to see some statistically significant separation in 3-D similarity scores between the partitioned noninactive-noninactive (NN) pairs and noninactive-inactive (NI) pairs. This requires 3-D similarity scores for both the NN pairs and NI pairs for each assay considered. This information is already available in the all-by-all similarity score matrices for the 734-K biologically tested molecules computed in the first part of this study. A detailed procedure for extracting the 3-D similarity scores from these matrices on the per-AID basis was described in the **Materials and Methods **section.

It is important to note that 3-D similarity methodologies (or other analysis methodologies, for that matter) are not expected to work for all biological assay data sets. A tacit assumption of 3-D methodologies is that chemical structures with similar shape and binding features will have similar (if not the same) mode of action of "activity", *e.g.*, of binding to a protein binding pocket in the same fashion. In reality, some assays in PubChem do not have a well-defined target, *e.g.*, being a whole cell, meaning that there could be a number of targets and a number of different mechanisms of action per target for the observed activity in a single assay. In other cases, many chemical structures are active for reasons that have little to do with binding to a protein target, being aggregators, covalent binders, cytotoxic, or some other unintended mode giving rise to the measured "activity" during the biological test (so called "false positives"). As such, 3-D methodology cannot be expected to work for false positives, as reputed "active" molecules may not have any apparent 3-D correlation to each other. This is also true of cases of molecules that would be "active" if not for solubility or some other issue during the biological experiment performed (so called "false negatives"). These issues with biological tests will be nearly completely ignored for the purpose of this analysis. Instead, by looking across a wide set of assays and assay types, there is an expectation that, if there is some effect whereby 3-D similarity averages between "actives" will be greater than the averages between "actives" and "inactives" using a single conformer per compound, a certain subpopulation of assays will show this behavior.

#### C-1. Selection of AIDs from the PubChem BioAssay database

**Figure 8:**
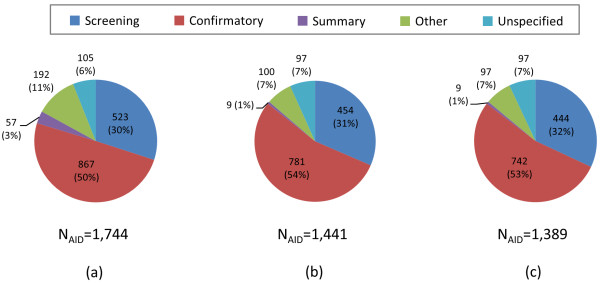
**Assay counts by category**. Assay count for each assay-type category in the PubChem BioAssay database: (a) for assays that have at least one tested molecule with 3-D information (as of January 28, 2010), (b) for assays that have at least one noninactive-noninactive (NN) pair and one noninactive-inactive (NI) pair, and (c) for assays that have at least six NN pairs and six NI pairs.

Among the 2,008 AIDs archived in the PubChem BioAssay database at the time of project initiation (January 2010), 1,744 AIDs had at least one molecule with a 3-D theoretical description. The bioassays in the PubChem BioAssay database can be classified into four categories, according to user-provided assay types (*i.e.*, screening, confirmatory, summary, and other) and the assay count for each category in the 1,744 AIDs is shown in Figure 8 (a). Note that there is another category, "Unspecified", because the assay-type attribute for these AID records are not provided. There were 523 screening assays (30%), 867 confirmatory assays (50%), 57 summary assays (3%), 192 other assays (11%), and 105 unspecified (6%).

For a given AID, comparison of the 3-D similarity scores for the NN pairs with those for the NI pairs requires that the AID has at least one NN pair and one NI pair. Among the 1,744 AIDs, there were 1,441 AIDs that satisfy this condition [Figure 8 (b)]. Further filtering was necessary to remove AIDs in which the number of NN or NI pairs is too small, because these AIDs may yield biased results. On the contrary, we did not want to filter out more summary assays, if it could be avoided, as there were only nine summary assays at this point. [Summary assays are final stages of lead/probe screening processes and, as such, they have a significantly smaller number of molecules provided (and hence, a smaller number of the NN and NI pairs), compared to other assay types.] Among the nine summary assays in Figure 8 (b), AID 1844 had the smallest number of the NN pairs, which was six, and this number was used as a threshold for further filtering (*i.e.*, AIDs with less than six NN pairs or less than six NI pairs were excluded in any subsequent analysis). After requiring an assay to have a minimum of six compound pairs for each of the NN and NI pairs (that is, 12 pairs per-AID in total), 1,389 AIDs resulted. As shown in Figure 8 (c), there were 444 primary screenings (32%), 742 confirmatory screenings (53%), 9 summary assays (1%), 97 other assays (7%), and 97 unspecified (7%).

#### C-2. Differences between the 3-D similarity scores of NN and NI pairs

**Figure 9:**
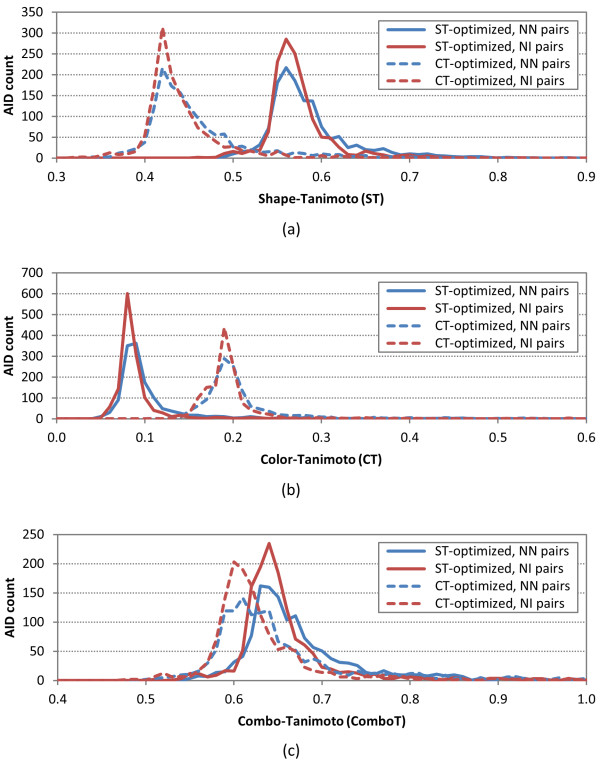
***μ*(*XT*) per-AID similarity histogram**. The distribution of the average similarity scores for noninactive-noninactive (NN) pairs and noninactive-inactive (NI) pairs of 1,389 AIDs in the PubChem BioAssay database: (a) shape-Tanimoto (ST), (b) color-Tanimoto (CT), and (c) Combo-Tanimoto (ComboT). All values binned in 0.01 increments.

**Figure 10:**
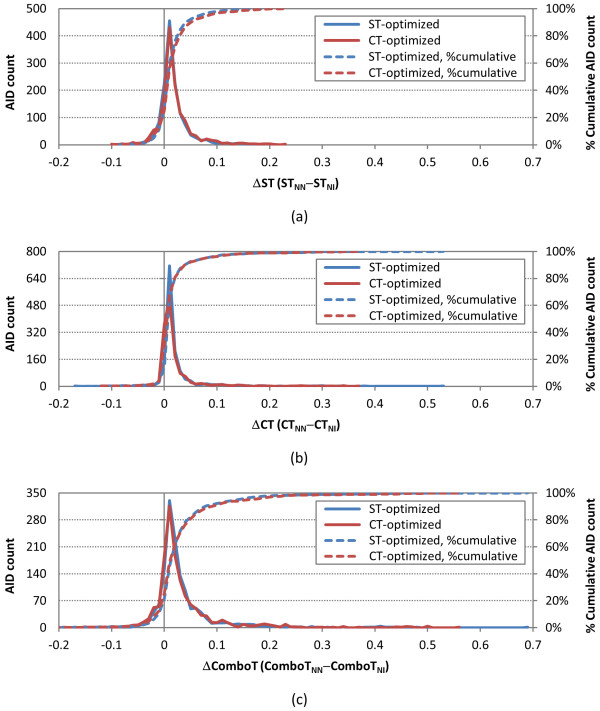
***μ*(*XT***_***NN-NI***_**) per-AID similarity statistics**. The distribution of the difference of the average similarity scores for noninactive-noninactive (NN) pairs and noninactive-inactive (NI) pairs of 1,389 AIDs in the PubChem BioAssay database: (a) shape-Tanimoto (ST), (b) color-Tanimoto (CT), and (c) Combo-Tanimoto (ComboT). All values binned in 0.01 increments.

With the set of 1,389 AIDs decided, the average and standard deviation [*i.e.*, *μ*(*XT*) and *σ*(*XT*), respectively] of the six different similarity values were determined for the NN and NI pairs per-AID. The complete set of per-AID results is available in Additional File 1, and the distributions of the per-AID average similarity scores for the NN and NI pairs [*i.e.*, *μ*(*XT*_*NN*_) and *μ*(*XT*_*NI*_), respectively] across the 1,389 AIDs are shown in Figure 9. The corresponding distributions of differences between the average similarity scores for NN and NI pairs per-AID [*i.e.*, *μ*(*XT*_*NN-NI*_)] are provided in Figure 10, while Table 3 and Table 4 summarize by similarity optimization type the per-AID statistics across all 1,389 AIDs [*i.e.*,*μ*[*μ*(*XT)*], *σ*[*μ*(*XT*)],*μ*[*σ*(*XT*)] and *σ*[*σ*(*XT*)]], with further break out by assay type category.

**Table 3:** Summary statistics per-AID for shape-Tanimoto (ST) optimized 3-D similarity.

	**XT=ST**^***ST-opt***^			**XT=CT**^***ST-opt***^			**XT=ComboT**^***ST-opt***^		
	
	Y=NN	Y=NI	Y=NN-NI	Y=NN	Y=NI	Y=NN-NI	Y=NN	Y=NI	Y=NN-NI
**μ[μ(XT**_**Y**_**)]**									
Screening	0.58	0.57	0.01	0.09	0.09	0.01	0.67	0.65	0.02
Confirmatory	0.58	0.57	0.01	0.11	0.09	0.02	0.70	0.66	0.03
Summary	0.67	0.64	0.03	0.35	0.25	0.10	1.01	0.88	0.13
Other	0.58	0.56	0.02	0.11	0.09	0.03	0.69	0.64	0.05
Unspecified	0.56	0.56	0.00	0.07	0.07	0.00	0.63	0.62	0.00
All	0.58	0.57	0.01	0.11	0.09	0.02	0.69	0.66	0.03
									
**σ[μ(XT**_**Y**_**)]**									
Screening	0.04	0.03	0.02	0.04	0.05	0.02	0.07	0.07	0.04
Confirmatory	0.05	0.04	0.03	0.08	0.05	0.05	0.12	0.08	0.07
Summary	0.13	0.10	0.05	0.26	0.19	0.17	0.39	0.29	0.22
Other	0.06	0.05	0.03	0.06	0.04	0.04	0.10	0.09	0.06
Unspecified	0.03	0.02	0.02	0.02	0.01	0.01	0.04	0.03	0.03
All	0.05	0.04	0.03	0.07	0.05	0.04	0.11	0.08	0.06
									
**μ[σ(XT**_**Y**_**)]**									
Screening	0.10	0.09	0.00	0.07	0.06	0.00	0.15	0.13	0.01
Confirmatory	0.11	0.11	0.01	0.10	0.07	0.01	0.19	0.16	0.01
Summary	0.12	0.11	0.02	0.19	0.17	0.04	0.30	0.26	0.06
Other	0.11	0.10	0.01	0.11	0.08	0.01	0.20	0.15	0.01
Unspecified	0.14	0.14	0.01	0.08	0.07	0.00	0.19	0.18	0.01
All	0.11	0.10	0.01	0.09	0.07	0.01	0.17	0.15	0.01
									
**σ[σ(XT**_**Y**_**)]**									
Screening	0.01	0.01	0.01	0.04	0.03	0.01	0.05	0.04	0.02
Confirmatory	0.02	0.03	0.01	0.06	0.04	0.02	0.07	0.06	0.02
Summary	0.05	0.03	0.03	0.13	0.09	0.06	0.18	0.11	0.09
Other	0.03	0.03	0.01	0.07	0.05	0.02	0.08	0.07	0.02
Unspecified	0.02	0.02	0.01	0.03	0.02	0.00	0.03	0.02	0.01
All	0.02	0.03	0.01	0.06	0.04	0.02	0.07	0.05	0.02

The overall average (μ) and standard deviation (σ) of the AID-specific average and standard deviation for noninactive-noninactive (NN) pairs and noninactive-inactive (NI) pairs of 1,389 AIDs in the PubChem BioAssay database.

**Table 4:** Summary statistics per-AID for color-Tanimoto (CT) optimized 3-D similarity.

	**XT=ST**^***CT-opt***^			**XT=CT**^***CT-opt***^			**XT=ComboT**^***CT-opt***^		
	
	Y=NN	Y=NI	Y=NN-NI	Y=NN	Y=NI	Y=NN-NI	Y=NN	Y=NI	Y=NN-NI
**μ[μ(XT**_**Y**_**)]**									
Screening	0.44	0.43	0.01	0.20	0.19	0.01	0.64	0.62	0.02
Confirmatory	0.46	0.44	0.02	0.21	0.20	0.02	0.67	0.64	0.04
Summary	0.57	0.54	0.03	0.43	0.37	0.07	1.00	0.90	0.10
Other	0.46	0.43	0.03	0.23	0.20	0.03	0.68	0.62	0.06
Unspecified	0.44	0.44	0.00	0.17	0.16	0.00	0.61	0.60	0.00
All	0.45	0.44	0.02	0.21	0.19	0.01	0.66	0.63	0.03
									
**σ[μ(XT**_**Y**_**)]**									
Screening	0.05	0.03	0.03	0.04	0.04	0.02	0.08	0.07	0.05
Confirmatory	0.07	0.05	0.04	0.08	0.05	0.05	0.14	0.09	0.08
Summary	0.18	0.14	0.07	0.26	0.19	0.10	0.43	0.33	0.15
Other	0.07	0.07	0.04	0.07	0.06	0.04	0.13	0.12	0.08
Unspecified	0.03	0.02	0.03	0.02	0.02	0.02	0.05	0.03	0.04
All	0.06	0.05	0.04	0.07	0.05	0.04	0.13	0.09	0.07
									
**μ[σ(XT**_**Y**_**)]**									
Screening	0.12	0.12	0.00	0.08	0.06	0.00	0.17	0.15	0.01
Confirmatory	0.13	0.13	0.01	0.10	0.08	0.01	0.21	0.18	0.01
Summary	0.16	0.15	0.03	0.17	0.16	0.04	0.32	0.29	0.07
Other	0.13	0.12	0.01	0.11	0.08	0.01	0.22	0.17	0.01
Unspecified	0.15	0.14	0.01	0.10	0.09	0.00	0.21	0.20	0.01
All	0.13	0.13	0.01	0.09	0.08	0.01	0.20	0.17	0.01
									
**σ[σ(XT**_**Y**_**)]**									
Screening	0.02	0.01	0.01	0.03	0.02	0.01	0.05	0.04	0.02
Confirmatory	0.03	0.02	0.01	0.05	0.04	0.02	0.07	0.06	0.03
Summary	0.08	0.05	0.05	0.11	0.08	0.05	0.19	0.12	0.10
Other	0.03	0.03	0.01	0.06	0.04	0.01	0.08	0.07	0.02
Unspecified	0.02	0.01	0.01	0.03	0.02	0.00	0.04	0.02	0.01
All	0.03	0.02	0.01	0.05	0.04	0.01	0.07	0.05	0.03

The overall average (μ) and standard deviation (σ) of the AID-specific average and standard deviation for noninactive-noninactive (NN) pairs and noninactive-inactive (NI) pairs of 1,389 AIDs in the PubChem BioAssay database.

When looking at the distributions in Figure 9 of the per-AID results, it is interesting to see, for a single conformer per compound anyway, that the per-AID average similarity distribution of NN pairs (primarily corresponding to the reputed "active/active" compound space) overlaps extensively with those of the NI pairs (essentially the reputed "active/inactive" compound space). The original hope was that there might be two clearly separated distributions, as this would be a clear signal that 3-D similarity using a single conformer per compound is able to distinguish between "actives" and "inactives" across all PubChem assays, but this is clearly not the case. The average and standard deviation of the  and  values per-AID over the 1,389 AIDs in Table 3 were 0.58 ± 0.05 and 0.57 ± 0.04, respectively. The corresponding values for  and  were 0.11 ± 0.07 and 0.09 ± 0.05, respectively. The small differences in these overall averages between the NN and NI pairs per-AID should *not *be considered statistically significant, considering their standard deviations. In fact, the average of averages per-AID for the NN and NI pairs are not significantly different from the ST^ST-opt ^and CT^ST-opt ^values for random conformers (0.54 ± 0.10 and 0.07 ± 0.05, respectively), listed in Table 2. For the same reason, the *ComboT*^*ST-opt *^differences between the NN and NI pairs are also not statistically significant. Note that, although the  values primarily increase from primary screenings to confirmatory assays to summary assays in general, this increase should also *not *be interpreted to be statistically meaningful, considering that the  values increases even more rapidly, as shown in Tables 3-4. The optimization type (*i.e.*, either ST- or CT-optimization) was also found to not make significant difference in *μ*(*XT*_*NN-NI*_) values.

Despite the significant overlap between the distributions for the NN and NI pairs in Figure 9, there are very subtle differences between them; for all six similarity scores, the NN-pair distributions, compared to the NI-pair distributions, have smaller AID counts at the peak and greater AID counts at the upper-tail region, indicating a small shift of the NN-pair distribution toward high similarity scores. This shift is also reflected in sharp, (mostly) normal distributions of *μ*(*XT*_*NN-NI*_), centered on the positive side just above zero in all cases (Figure 10). This suggests that single conformer per compound 3-D similarity is showing some of the anticipated effect of the "similarity principle", which states that structurally similar molecules are likely to have similar biological activities [33-36], such that the "active/active" space is separated from the "active/inactive" space; however, for most assays in PubChem, this effect is simply not large enough to be unambiguous for all biological assays, as reflected in the μ[μ(XT_NN-NI_)] values smaller than σ[μ(XT_NN-NI_)] for all six similarity measures. Tables 3 and 4 also clearly show that, in general, there is no clear statistically meaningful separation across assays or assay category type using a single conformer per compound. For example, while there is clearly a positive average of NN-NI difference across all similarity score types for all assays and all assay categories, ranging from 0.00-0.13 for μ[μ(XT_NN-NI_)], the corresponding standard deviation of the average [*i.e.*, "σ[μ(XT_NN-NI_)]"] is consistently larger than the average value.

These results lead to a number of questions. Why isn't there a greater, unambiguous separation in the 3-D similarity scores between the NN and NI pairs? Is it that we are employing a single conformer per compound in the analysis? After all, the current PubChem3D theoretical conformer generation approach does not guarantee that the single (default) conformer used for each molecule in the NN pairs is a (or "the") bioactive conformation. A general premise of the interpretation of 3-D similarity between a NN pair requires a "bioactive" conformation surrogate for *both *noninactive molecules. Estimating 3-D similarity between "non-bioactive" conformers of both molecules, or between a "bioactive" conformer of one molecule and a "non-bioactive" conformer of the other, is essentially identical to 3-D similarity comparison for the NI pairs. Therefore, the use of a single conformer per compound is not likely to result in enough similarity score difference between the NN and NI pairs across a wide set of assays. Using multiple conformers per compound may result in a greater separation in similarity scores between the NN and NI pairs, but performing the same analysis using multiple conformers per compound is prohibitively expensive, considering that we are dealing with 269.7 billion conformer pairs arising from 734 thousand compounds and optimizing each conformer pair by ST and then by CT (9 TB of data gzip compressed). Any increase in the count of conformers also increases the computational complexity (and data storage requirements) by the square of the number of conformers per compound considered.

From a gross statistical approach, there is not sufficient separation across the averages of assays for a single conformer per compound to say definitively there is a clear separation between NN and NI pairs. It could be that, by considering multiple conformers per compound (and picking the best similarity conformer pair per compound pair), a clearer separation may occur, but this is a study for another day (and a bigger computer cluster and a bigger data storage system). There are, however, clear examples where some AIDs do show a clear separation, as shown in the tail regions of Figure 10, using only a single conformer per compound.

#### C-3. Outliers

**Figure 11:**
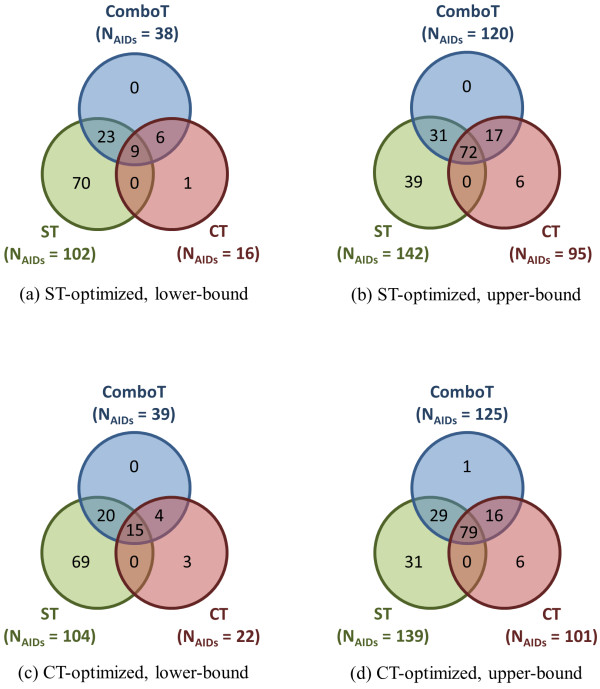
**Assay *μ*[*μ*(*XT***_***NN-NI***_**)] outlier commonality by 3-D similarity type**. The Venn diagrams show the number of AIDs whose difference of the average similarity scores for noninactive-noninactive (NN) pairs and noninactive-inactive (NI) pairs of 1,389 AIDs in the PubChem BioAssay database are out of the range of , where "lower-bound" corresponds to μ - σ and "upper-bound" corresponds to μ + σ.

Although the overall average differences in similarity scores between the NN and NI pairs were not statistically significant, some AIDs do have substantial (and statistically meaningful) NN-NI differences. These "outlier" cases correspond to the tail regions of the distribution curves in Figure 10. For each of the six similarity measures, the AIDs that lie outside the  region were extracted and are henceforth defined as "outliers". Figure 11 shows Venn diagrams detailing the outlier overlap as a function of 3-D similarity score type. To aid in discussion, the AIDs that have a statistically significant positive value of average NN-NI difference are deemed "upper-bound" cases [Figure 11(b) and 11(d)] and the AIDs that have a statistically significant negative value of average NN-NI difference are deemed "lower-bound" cases [Figure 11(a) and 11(c)].

The lower-bound cases are when the average 3-D similarity scores for "active/inactive" compound pairs are greater than for "active/active" compound pairs, a counter result to the whole notion of chemical similarity. While the opposite of what one might expect, it can readily occur from a set of chemical structures that are predominately 3-D similar, being on both sides of that subjective and (at times) arbitrary line of being "active" or "inactive", and where most compounds in the compound series are considered "inactive", as can be the case with well defined "activity cliffs" [34,37-40].

Among the 109 unique, lower-bound  outliers, 102 (94%) are  outliers whereas only 7 (6%) are unique to  outliers [Figure 11 (a)]. A similar trend is found in the case of lower-bound  outliers [Figure 11 (c)]. Perhaps this should not be a surprise as shape alone (ignoring features) might not be expected to be a good discriminator of "actives" and "inactives". On the other hand, as shown in Figure 11 (b), there are relatively few unique upper-bound outlier cases solely attributable to  and , being only 39 (24%) and 6 (4%), respectively, of the total. Rather, there is significant overlap between all three 3-D similarity score types, , , and , with 120 of the 165 (73%) unique upper-bound outlier cases common to . Again, a similar trend is found for upper-bound  outliers [Figure 11 (d)]. This suggests, for the upper-bound AID outlier cases, use of ComboT similarity score is most efficient at finding most of the outlier cases when using a single conformer per compound.

**Figure 12:**
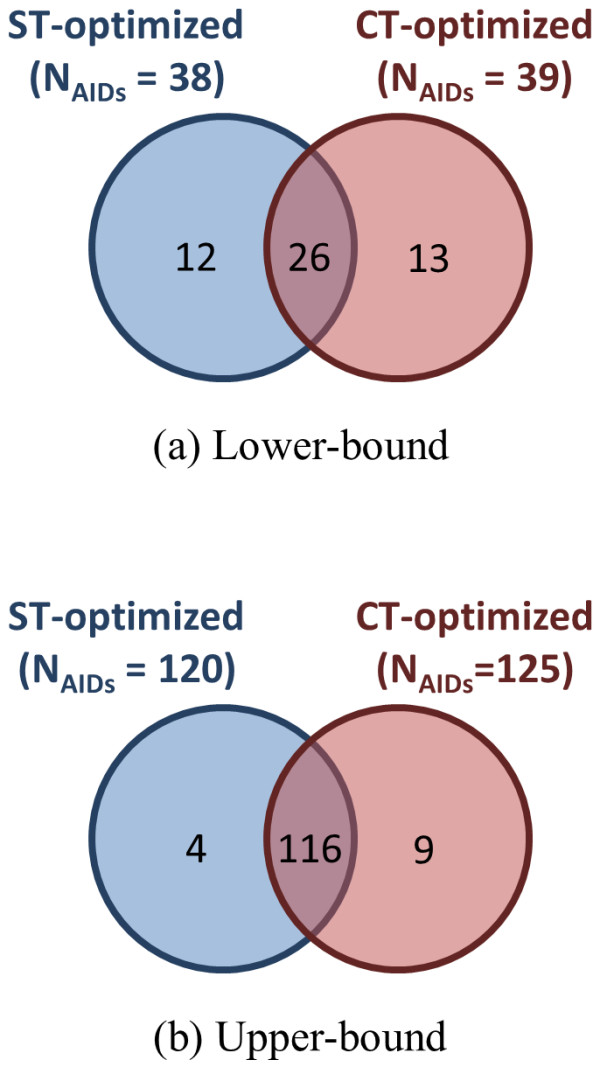
**Assay *μ*[*μ*(*ComboT***_***NN-NI***_**)] outlier commonality by superposition optimization type**. The Venn diagrams show the number of AIDs whose difference of the average ComboT similarity scores for noninactive-noninactive (NN) pairs and noninactive-inactive (NI) pairs of 1,389 AIDs in the PubChem BioAssay database that are out of the range of , where "lower-bound" corresponds to μ - σ and "upper-bound" corresponds to μ + σ. Upper-bound outliers tend to be shared by both superposition optimization types, while lower-bound outliers are less shared.

Figure 12 compares the  and  AID outlier cases. There are 120 and 125 upper-bound AID outliers for  and , respectively, and 116 are common to both. In contrast, there are 26 AIDs common in the  and  lower-bound outliers, while about half that value are unique to each. This shows that the upper-bound AID outliers are predominately conformer superposition optimization type independent.

Table 5 gives the top 25% of the common *ComboT*_*NN-NI *_upper-bound AID outliers, yielding the largest magnitude difference in average NN-NI separation, and Table 6 gives all common *ComboT*_*NN-NI *_lower-bound AID outliers. Table 7 lists the count of assay outliers broken down by optimization type and similarity metric type. Exploring the top five assays in Table 5, the first three represent trivial examples of a compound series easily identifiable using 2-D similarity or 3-D similarity or by eye. AID 672, with the fourth largest NN-NI positive difference found, is somewhat more interesting.

**Table 5:** Top 25% of common upper-bound assay *μ*(*ComboT*_*NN-*__*N*__*I*_) outliers.

		**μ(ComboT**_**NN-NI**_**)**			
AID	Type *^a^*				Description
		ST-opt	CT-opt	**Ave.**^***a***^	
1475	(S)	0.68	0.46	0.57	Quantitative High-Throughput Screen for Inhibitors of Tau Fibril Formation: Summary
2163	(C)	0.47	0.50	0.49	Cuvette-Based Assay for Inhibitors of 12-hLO (12-human lipoxygenase)
838	(C)	0.41	0.56	0.48	Mycobacterium tuberculosis Pantothenate Synthetase Secondary Assay
**672**	**(C)**	**0.45**	**0.50**	**0.48**	**Redox Cycling Hydrogen Peroxide Generation Assay; MKP-1 and MKP-3 Probe Assessment**.
**2230**	**(C)**	**0.41**	**0.49**	**0.45**	**Confirmation assay for inhibitors of Trypanosoma brucei hexokinase 1-Analogue-first series**
653	(C)	0.44	0.46	0.45	West Nile Virus NS2bNS3 Proteinase Inhibitor Dose Response Confirmation.
766	(C)	0.42	0.45	0.43	Formylpeptide Receptor (FPRL1) Ligand Structure Activity Relationship (SAR) Analysis : Dose Response Assay
486	(C)	0.43	0.44	0.43	Clearance of Mutant Huntingtin Protein - Confirmatory screen
2267	(C)	0.40	0.46	0.43	Secondary assay for Inhibitors of Human Pyruvate Kinase M2 isoform
1068	(C)	0.39	0.47	0.43	BAP1 Enzyme inhibitors AMC/RHO Comparison
523	(C)	0.40	0.44	0.42	Cathepsin B Inhibitor Series SAR Study
1536	(C)	0.34	0.49	0.42	Confirmation of compounds inhibiting phosphomannose isomerase (PMI) via a fluorescence intensity assay using a high concentration of mannose 6-phosphate.
346	(C)	0.37	0.45	0.41	HIV Nucleocapsid
1083	(C)	0.40	0.41	0.40	Concentration Response fluorescence polarization-based assay to test purchased Analogs of Selected Hits from the Polo box domain (PBD) of Plk1 Primary HTS.
732	(P)	0.34	0.34	0.34	In Vivo Angiogenesis Assay for HTS
718	(C)	0.31	0.32	0.31	Dose Response assay for agonists of 5-Hydroxytryptamine (Serotonin) Receptor Subtype 1A (5HT1A)
1157	(O)	0.26	0.36	0.31	PSACAntagonistScreen(Absorb.700[IndoChina.Blood(.18h)])
1315	(P)	0.28	0.34	0.31	Fluorescence polarization for PKD inhibitors - interference assay (140K library campaign)
1781	(C)	0.27	0.34	0.31	Confirmation Concentration-Response Assay for Activators of Human Liver Pyruvate Kinase
579	(C)	0.26	0.26	0.26	In vitro MKP-1 Phosphatase Dose Response SAR Support Assay
1039	(C)	0.20	0.30	0.25	Normal 2 Cell Viability Secondary Assay for qHTS Assay for Epigenetic Modulators
1540	(C)	0.27	0.23	0.25	Secondary assay for Activators of Human Pyruvate Kinase M2 isoform
835	(C)	0.20	0.29	0.24	Dose-response biochemical assay for antagonists of the interaction between the Eph receptor B4 (EphB4) and its ligand ephrin-B2 via TNYL-RAW peptide
621	(C)	0.24	0.23	0.24	TR-FRET secondary assay for HTS discovery of chemical inhibitors of anti-apoptotic protein Bfl-1
1751	(C)	0.23	0.23	0.23	Confirmation Concentration-Response Assay for Activators of Human Muscle isoform 2 Pyruvate Kinase
254	(?)	0.19	0.25	0.22	NCI In Vivo Anticancer Drug Screen. Data for tumor model L1210 Leukemia (intravenous) in B6D2F1 (BDF1) mice
552	(P)	0.24	0.19	0.22	Antimicrobial HTS Assay for E. coli BW25113 (wild type)
734	(P)	0.20	0.23	0.22	Assay to identify inhibitors among the possible fluorescent artifacts from the primary HTS inhibition assay of Matrix Metalloproteinase 13 (MMP13) activity

A list of AIDs whose difference in the average ComboT similarity scores between noninactive-noninactive (NN) pairs and noninactive-inactive (NI) pairs is greater than μ[μ(ComboT_NN-NI_)] + σ[μ(ComboT_NN-NI_)] for both superposition optimization types.

^*a *^(P), (primary) screening; (C), confirmatory; (S), summary; (O), others; (?), unspecified.

^*b *^The average of ST-optimized and CT-optimized ComboT_NN-NI_.

**Table 6:** Common lower-bound assay *μ*(*ComboT*_*NN-*__*N*__*I*_) outliers.

		**μ(ComboT**_**NN-NI**_**)**			
AID	Type *^a^*				Description
		ST-opt	CT-opt	**Ave.**^***b***^	
1349	(P)	-0.27	-0.22	-0.24	Test of compound activity against tunicamycin-induced cell death using Flow cytometry in CSM14.1 undifferentiated cells.
1343	(P)	-0.18	-0.20	-0.19	Test of compound activity against tunicamycin-induced cell death using an ATP content assay in CSM14.1 undifferentiated cells.
1218	(P)	-0.11	-0.16	-0.13	Functional assay for estrogen-mediated translocation of PIP3: Secondary Assay for Estrogen receptor beta.
1219	(P)	-0.11	-0.16	-0.13	Functional assay to assess estrogen-mediated translocation of PIP3: Secondary Assay for GPR30 estrogen receptor.
1711	(C)	-0.12	-0.13	-0.13	Concentration-Response Counterscreen for Tau: Redox Active Inhibitors of Caspase-1
1247	(C)	-0.14	-0.10	-0.12	Dose Response Assay for SAR studies of Inhibitors of ER stress-Induced Cell Death: A Benzodiazepine Series
839	(C)	-0.09	-0.12	-0.10	Screening for Modulators of Post-Golgi Transport - Secondary Growth Assay.
495	(O)	-0.07	-0.14	-0.10	Literature data for small-molecule inhibitors of Influenza A Subtype N2.
931	(C)	-0.10	-0.11	-0.10	Confirmation and Secondary Assay for Modulators of Hemoglobin Beta Chain Splicing at IVS2 654 locus: Cytotoxicity
791	(C)	-0.10	-0.08	-0.09	Dose-response biochemical assay of Rho kinase 2 (Rock2) inhibitors
769	(C)	-0.09	-0.07	-0.08	Dose response biochemical assay for autofluorescent inhibitors of Matrix Metalloproteinase 13 (MMP13) activity
302	(?)	-0.06	-0.08	-0.07	NCI In Vivo Anticancer Drug Screen. Data for tumor model Madison 109 Lung Carcinoma (intramuscular) in BALB/CM mice
1225	(C)	-0.05	-0.07	-0.06	Dose Response cell-based high-throughput screening assay to identify antagonists of galanin receptor 2 (GALR2)
1226	(P)	-0.05	-0.07	-0.06	Functional assay for estrogen-mediated translocation of PIP3: Secondary Assay for Estrogen receptor alpha
2110	(C)	-0.05	-0.07	-0.06	Confirmation of Inhibitors and Activators of Purified Human alpha-Glucosidase Using an Alternate Red Fluorescent Susbtrate
1331	(C)	-0.04	-0.08	-0.06	Dose response biochemical High Throughput Screening assay for agonists of the steroid receptor coactivator 3 (SRC-3) recruitment by the peroxisome proliferator-activated receptor gamma (PPARgamma)
324	(?)	-0.06	-0.05	-0.06	NCI In Vivo Anticancer Drug Screen. Data for tumor model P388 Leukemia resistant to AMSA; NSC 249992 (intraperitoneal) in CD2F1 (CDF1) mice
1342	(C)	-0.05	-0.06	-0.06	Fluorescence polarization assay for PKD inhibitiors-interference assay-57K HTS campaign
308	(?)	-0.05	-0.05	-0.05	NCI In Vivo Anticancer Drug Screen. Data for tumor model L1210 Leukemia resistant to 6-MP and 6-Thioguanine; NSC 755, NSC 752 (intraperitoneal) in B6D2F1 (BDF1) mice
1659	(C)	-0.04	-0.06	-0.05	SAR assay for compounds activating TNAP in the presence of 100 mM DEA performed in a luminescence assay
658	(C)	-0.04	-0.06	-0.05	Cellular Toxicity (caspase-3) BJ
632	(C)	-0.04	-0.06	-0.05	Confirmation Concentration-Response Assay and Counterscreen for Disrupters of an Hsp90 Co-Chaperone Interaction
238	(?)	-0.05	-0.04	-0.04	NCI In Vivo Anticancer Drug Screen. Data for tumor model Leiomyosarcoma (No. 2) (intraperitoneal) in CAF1 mice
856	(C)	-0.04	-0.05	-0.04	Counterscreen for S1P2 Antagonists: Dose Response Cell-Based Screen to Identify Antagonists of CRE-BLA
232	(?)	-0.04	-0.05	-0.04	NCI In Vivo Anticancer Drug Screen. Data for tumor model Lymphosarcoma Gardner 6C3HED (intraperitoneal) in C3AKF1 (CHKRF1) mice
369	(O)	-0.04	-0.04	-0.04	Literature data for small-molecule inhibitors of Avian Sarcoma Virus Src.

A list of AIDs whose difference in the average ComboT similarity scores between noninactive-noninactive (NN) pairs and noninactive-inactive (NI) pairs is greater in magnitude than μ[μ(ComboT_NN-NI_)] - σ[μ(ComboT_NN-NI_)] for both superposition optimization types.

^*a *^(P), (primary) screening; (C), confirmatory; (S), summary; (O), others; (?), unspecified.

^*b *^The average of ST-optimized and CT-optimized ComboT_NN-NI_.

**Table 7:** Outlier breakdown by optimization type and similarity type.

	Lower-bound (< μ[μ(XT)] - σ[μ(XT)])			Upper-bound (> μ[μ(XT)] + σ[μ(XT)])		
	
	ST	CT	ComboT	ST	CT	ComboT
**ST-optimization**						
screening	10 (2.3%)	7 (1.6%)	7 (1.6%)	32 (7.2%)	11 (2.5%)	23 (5.2%)
confirmatory	69 (9.3%)	8 (1.1%)	19 (2.6%)	85 (11.5%)	63 (8.5%)	75 (10.1%)
summary	1 (11.1%)	0 (0.0%)	0 (0.0%)	2 (22.2%)	3 (33.3%)	2 (22.2%)
other	4 (4.1%)	1 (1.0%)	3 (3.1%)	19 (19.6%)	17 (17.5%)	19 (19.6%)
unspecified	18 (18.6%)	0 (0.0%)	9 (9.3%)	4 (4.1%)	1 (1.0%)	1 (1.0%)
All	102 (7.3%)	16 (1.2%)	38 (2.7%)	142 (10.2%)	95 (6.8%)	120 (8.6%)
						
**CT-optimization**						
screening	10 (2.3%)	8 (1.8%)	9 (2.0%)	31 (7.0%)	13 (2.9%)	23 (5.2%)
confirmatory	70 (9.4%)	12 (1.6%)	22 (3.0%)	81 (10.9%)	66 (8.9%)	76 (10.2%)
summary	2 (22.2%)	0 (0.0%)	1 (11.1%)	2 (22.2%)	3 (33.3%)	3 (33.3%)
other	3 (3.1%)	2 (2.1%)	2 (2.1%)	22 (22.7%)	18 (18.6%)	21 (21.6%)
unspecified	19 (19.6%)	0 (0.0%)	5 (5.2%)	3 (3.1%)	1 (1.0%)	2 (2.1%)
All	104 (7.5%)	22 (1.6%)	39 (2.8%)	139 (10.0%)	101 (7.3%)	125 (9.0%)

The count of assay outliers that have a significant similarity score difference between the NN and NI pairs. Numbers in parentheses are the percentages of outliers relative to the assay-type counts shown in Figure 8(c).

**Figure 13:**
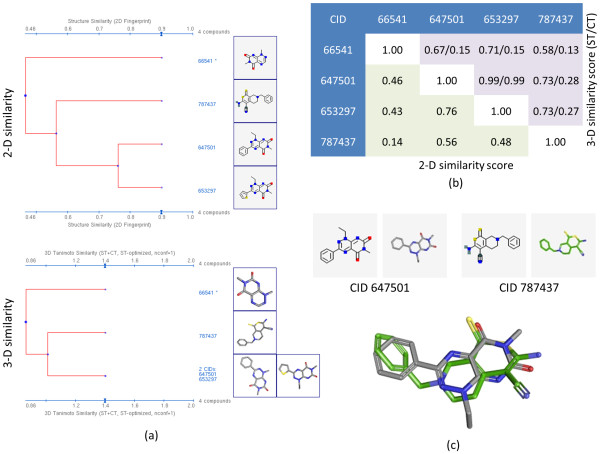
**Separation between actives and inactives**. An example of clear separation between  3-D similarities of 0.45 (see Table 5), the four active compounds from AID 672: (a) shows 2-D and 3-D similarity dendrograms generated using the PubChem Structure Clustering tool; (b) shows the respective 2-D similarity values (lower triangle) and 3-D similarity values (upper triangle); and (c) shows a representative alignment showing how CID 647501 is 3-D similar to CID 787437 (ST/CT 0.73/0.28), despite low 2-D similarity (0.56).

AID 672 is a secondary confirmatory assay with four active compounds, shown in Figure 13 (a), that comprise the NN pairs. Of these four structures, three have a similar substructure but only two of the structures (CIDs 647501 and 653297) might be considered "similar" with a 0.76 2-D similarity using the PubChem subgraph fingerprint [Figure 13 (b)]; however, using ComboT^ST-opt ^3-D similarity, all four compounds have pair-wise similarity beyond random (*i.e.*, ComboT^ST-opt ^> { μ + σ } = 0.74 from Table 2) except for one compound pair (CIDs 66541 and 787437). An example of one of these pair-wise superpositions [Figure 13 (c)] shows one way these different chemical structures can be superimposed relative to their shape and feature complements. While a relatively small example, and easy to examine in detail, there readily exists much larger examples.

**Figure 14:**
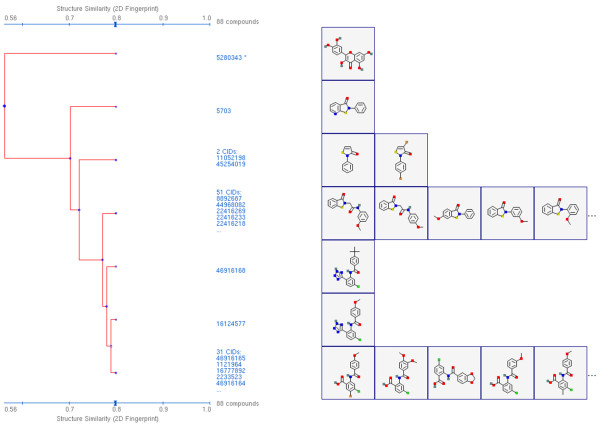
**2-D similarity isolates related chemical series**. Dendrogram from the PubChem Structure Clustering tool for 88 of the 92 noninactive pairs from AID 2230 showing two primary clusters (containing 51 and 31 compounds, respectively) at 0.8 Tanimoto using 2-D similarity. Note that all but one compound is related above 0.7 Tanimoto.

**Figure 15:**
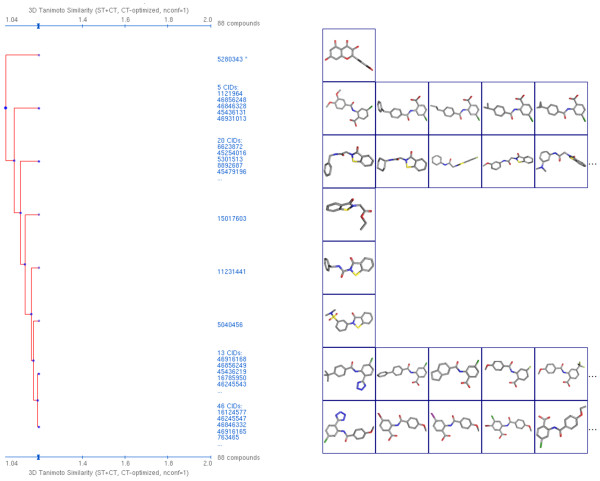
**3-D similarity interrelates chemical series**. Dendrogram from the PubChem Structure Clustering tool for 88 of the 92 noninactive pairs from AID 2230 showing three primary clusters (containing 46, 20, and 13 compounds, respectively) at 1.2 combo Tanimoto (ComboT) using 3-D similarity, CT-optimized. All structures are interrelated at a ComboT of 1.04, more than 3.2 standard deviations beyond the random pair average of 0.59.

AID 2230, also a secondary confirmatory assay and fifth in the list found in Table 5, possesses a much larger NN set with 92 compounds. When examining these by 2-D cluster analysis using the PubChem Structure Clustering tool, as shown in Figure 14, there are clearly two compound series, one with 51 compounds and the other with 31 compounds, representing the majority of the "active" chemical structures. Switching to 3-D ComboT similarity, all but four of the 92 compounds, as shown in Figure 15, are inter-related at a *ComboT*^*CT-opt *^value above 1.04. As shown in Table 2, a value of 1.04 is more than three standard deviations away from the random average of 0.59 for *ComboT*^*CT-opt*^. As one goes to a *ComboT*^*CT-opt *^value of 1.2, several different clusters appear with the largest containing 46 compounds and second largest containing 20 compounds. This demonstrates how 3-D similarity is able to relate chemical series distinct in 2-D similarity, as representing similar shape and feature space even with a single conformer per compound.

## Conclusion

Six 3-D similarity measures (*ST*^ST-opt^, *CT*^ST-opt^, *ComboT*^ST-opt^, *ST*^CT-opt^, *CT*^CT-opt^, and *ComboT*^CT-opt^) in conjunction with 734,486 biologically tested compounds from PubChem were utilized to help answer the question: what is a biologically meaningful 3-D similarity score? The distribution of the six similarity measures for biologically tested compound pairs, resulting from computation of all-against-all similarity scores (269.7 billion unique conformer pairs), yielded an average and standard deviation for *ST*^ST-opt^, *CT*^ST-opt^, *ComboT*^ST-opt^, *ST*^CT-opt^, *CT*^CT-opt^, and *ComboT*^CT-opt ^of 0.54 ± 0.10, 0.07 ± 0.05, 0.62 ± 0.13, 0.41 ± 0.11, 0.18 ± 0.06, and 0.59 ± 0.14, respectively. These values represent valuable benchmarks for the 3-D similarity values provided by PubChem and those computed by some commercial software packages. One can now know when a statistically meaningful superposition between a conformer pair occurs, potentially helping to improve their ability to analyze bioactivity information.

This random distribution of biologically tested compounds was constructed using a single theoretical conformer per compound (the "default" conformer provided by PubChem). If one were to use multiple diverse conformers per compound and pick the best 3-D similarity score, the average random distribution values may well be higher (perhaps significantly so); however, if one considers the continuum of all similarity values produced in the use of multiple diverse conformers per compound to yield a similar random distribution values, the averages (and standard deviations) above may still be applicable or, perhaps, treated as a conservative lower bound result. Further study is clearly warranted using multiple diverse conformers per compound. This work is a critical first step covering a very wide corpus of chemical structures and biological assays and creating a statistical framework to build upon.

The second part of this study explored the question of whether it was possible to realize a statistically meaningful 3-D similarity value separation between reputed biological assay "inactives" and "actives". Using the terminology of noninactive-noninactive (NN) pairs and the noninactive-inactive (NI) pairs to represent comparison of the "active/active" and "active/inactive" spaces, respectively, each of the 1,389 biological assays were examined by their 3-D similarity score differences between the NN and NI pairs and analyzed across all assays and assay category types. Regardless of the optimization type employed (*i.e.*, either of ST- or CT-optimization), the overall average difference between the *μ*(*XT*_*NN*_) and *μ*(*XT*_*NI*_) values, while consistently positive (as hoped), were not statistically unambiguous after considering their large standard deviations. Similarly, an increase in the  values upon going from primary screenings to confirmatory assays to summary assays was also not statistically meaningful, due to an even more rapid increase in the  values.

The negligible difference in 3-D similarity between the NN and NI pairs may be due to employing a single conformer per compound in this study. Conceivably the 3-D similarity between two noninactive molecules should be evaluated using the "bioactive" conformer for each molecule, being the conformer giving rise to the observed biological activity; however, the single conformers per compound used in the present study are not guaranteed to be sufficiently similar to the bioactive conformers, and the average similarity scores per-AID for the NN pairs were not much different than those from the NI pairs. Considering the negligible difference in the 3-D similarity scores between the NN and NI pairs, it may not be appropriate to analyze bioassay data with a single conformer per compound in a general sense. With that said, there were a subset of biological assays where a clear separation between the NN and NI pairs were found. In addition, use of combo Tanimoto (ComboT) alone, independent of superposition optimization type, appears to be the most efficient 3-D score type in identifying these cases.

## Materials and methods

### 1. Datasets

At the time of project initiation (late January of 2010), there were 2,008 bioassays (unique identifier AID) deposited in the PubChem BioAssay database, ranging from AID 1 to AID 2310. Among the chemical structures tested in these assays, those with associated PubChem Compound records (unique identifier CID) with theoretical 3-D conformer models available [22] were considered in the present study. Note that the 3-D information is only available for CIDs that satisfy the following restrictions [22,23]:

(1) is a single covalent component.

(2) contains only organic [H, C, N, O, F, P, S, Cl, Br, and I] elements

(3) possess only typical bonding situation (*e.g.*, no hyper valent situations)

(4) not too big (*e.g.*, 50 non-hydrogen atoms or less) and not too flexible (*e.g.*, 15 effective rotors or less)

(5) have five undefined stereocenters or less

There are 734,486 CIDs satisfying the above conditions for the 2,008 AIDs. All data is accessible from the PubChem website (http://pubchem.ncbi.nlm.nih.gov). Bulk download of data is also available from the PubChem FTP site (ftp://ftp.ncbi.nlm.nih.gov/pubchem). The AIDs considered are provided in Additional File 1 with per-AID statistics of 3-D similarity scores for the NN and NI pairs.

### 2. Similarity Score Computation

In the first part of this study, the first diverse conformer [24] for each of the 734,486 CIDs were downloaded. A total of six different 3-D similarity scores were computed, resulting from three different similarity metrics computed for conformer pairs superpositions optimized in two different ways. The three similarity metrics are: shape Tanimoto [ST, **Equation (1)**], measuring the shape similarity; color Tanimoto [CT, **Equation (2)**], measuring the similarity of 3-D orientation of functional groups used to defined pharmacophores (specified simply as features); and combo Tanimoto (ComboT), the simple sum of ST and CT [**Equation (3)**]. The two conformer superposition methods used optimize: by shape similarity (ST-optimized), where conformer shape overlap is maximized; and feature similarity (CT-optimized), where conformer feature overlap is maximized. Feature definitions and all similarities were computed using the C++ Shape toolkit [28] from OpenEye Scientific Software, Inc.

There were a total of 269,734,474,855 conformer pair similarity sets from all possible unique combinations of the 734,486 conformers. Histograms of the computed similarity scores were generated after binning all similarity scores in 0.01 increments [using the C function "rint(float)"]. Note that we used only the first diverse conformer for each compound, being the PubChem default conformer. Considering the total size of data files (9.0 TB compressed, when storing only the two conformer IDs, the two similarity scores, the 3 × 3 rotational matrix, and translation vector per conformer pair computed), employing additional conformers per compound in this study would quickly overwhelm the available computational resources and disk space to consider.

**Figure 16:**
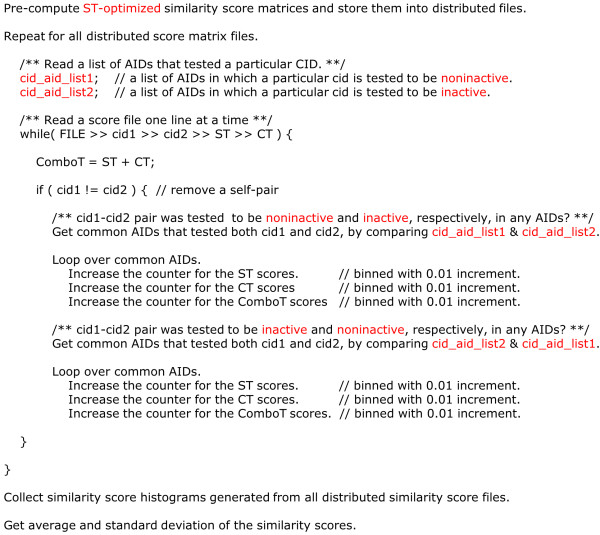
**Analysis method overview**. Pseudo code that describes the process by which the average and standard deviation of the ST-optimized similarity scores for noninactive-inactive (NI) pairs for individual bioassay were computed. This process was repeated for the CT-optimized similarity scores. For the average and standard deviation of the similarity scores for the noninactive-noninactive (NN) pairs were also computed in a similar manner, except that only the cid_aid_list1 (for noninactves) was searched both for cid1 and cid2.

Many of the compounds in the present study were biologically tested in multiple assays, and hence, a substantial fraction of conformer pairs appear in multiple assays. Therefore, since consideration is given to one assay at a time, extracting the similarity scores for the conformer pairs tested in each AID from the all-by-all similarity score matrices computed and stored in the first part of study is described in Figure 16.

## Competing interests

The authors declare that they have no competing interests.

## Authors' contributions

EEB computed the similarity score matrices. SK analyzed the data and wrote the first draft. SHB reviewed the final manuscript. All authors read and approved the final manuscript.

## Supplementary Material

Additional file 1 **Similarity Scores **Statistical parameters of similarity scores for each AID. Click here for file
